# Exploring the molecular mechanism of Er Miao San for treating rheumatoid arthritis based on network pharmacology

**DOI:** 10.1186/s12906-026-05359-6

**Published:** 2026-04-10

**Authors:** Zihua Xuan, Simeng Chen, Xiangwen Men, Jin Wang, Zhiluo Cheng, Min Liu, Jiayu Wang, Min Zhang, Xiaoyi Jia

**Affiliations:** 1 School of Pharmacy, Anhui University of Chinese Medicine, Hefei, 230012 China; 2 Anhui Province Key Laboratory of Bioactive Natural Products, Hefei, 230012 China; 3 Department of Rheumatology and Immunology, Division of Life Sciences and Medicine, The First Affiliated Hospital of USTC, University of Science and Technology of China, Hefei, 230012 China

**Keywords:** Rheumatoid arthritis, Traditional Chinese medicine, Network pharmacology, Molecular docking, NF-κB signaling pathway

## Abstract

**Objective:**

This study aimed to elucidate the molecular mechanism of Er Miao San (EMS) in treating rheumatoid arthritis (RA) by integrating network pharmacology, molecular docking, and experimental validation.

**Methods:**

Using public databases, potential targets of EMS, RA-related targets, and NF-κB pathway targets were cross-referenced to identify common genes. These were analyzed via protein-protein interaction (PPI)network, GO/KEGG enrichment analyses (via DAVID), and hub gene identification (using Cytoscape and cytoHubba). Core predictions were validated by molecular docking, with therapeutic efficacy confirmed in adjuvant-induced arthritis (AA)rats and NF-κB pathway inhibition confirmed in TNF-α-induced fibroblast-like synoviocytes (FLSs).

**Results:**

Integrated analysis identified 34 common targets of Er Miao San (EMS) for rheumatoid arthritis (RA) treatment, with NFKBIA, RELA, and TNF recognized as the top hub genes. Molecular docking revealed stable binding between these targets and the core active components (fumarine, berberine, and wogonin). Experimentally, EMS alleviated joint pathology in AA rats and concentration-dependently suppressed TNF-α-induced proliferation of FLSs and the secretion of IL-1β and IL-6. Mechanistically, EMS inhibited NF-κB signaling by reducing the phosphorylation of IκBα and p65 and blocking the nuclear translocation of p65.

**Conclusion:**

The therapeutic effects of EMS on RA are mediated through the inhibition of the NF-κB signaling pathway, thereby elucidating its mechanism of action.

**Supplementary Information:**

The online version contains supplementary material available at 10.1186/s12906-026-05359-6.

## Introduction

Rheumatoid arthritis (RA) is a systemic autoimmune disease whose aetiology remains incompletely understood, with chronic synovitis as its primary pathological hallmark [1, 2]. This condition exhibits progressive development, leading to persistent joint structural damage and ultimately functional impairment [3]. Although research indicates that both genetic and environmental factors play significant roles in its pathogenesis, the precise mechanisms underlying RA remain unclear [4]. Recent research evidence indicates that the NF-κB signalling pathway exerts a pivotal regulatory role in the onset and progression of RA. For instance, a study by Mao Jing et al. (2023) demonstrated that stimulation by neutrophil extracellular traps activates NF-κB pathway, thereby enhancing the transcription of pro-inflammatory cytokines and Caspase-3 genes in fibroblast-like synoviocytes (FLS) with RA [5]. The activation of NF-κB not only exacerbates the inflammatory response by promoting the release of inflammatory mediators such as IL-6, IL-1β, and TNF-α, but also interferes with the balance of proliferation and apoptosis of FLSs [6, 7]. Consequently, targeting the regulation of the NF-κB pathway is regarded as a potentially effective therapeutic strategy for RA, and exploring novel drugs that modulate this signaling pathway holds significant research value [8].

Traditional Chinese medicine (TCM), with its multi-component, multi-target action mode and excellent safety profile, offers a rich resource base for the development of new anti-RA drugs [9]. Er Miao San (EMS) is a classic TCM formula originally recorded by Zhu Danxi in the Yuan dynasty. This formula consists of two herbs in equal proportion: Atractylodis Rhizoma (Cangzhu), which is the rhizome of Atractylodes lancea (Thunb.) DC. (Compositae) and Phellodendri Cortex (Huangbai), which is the bark of Phellodendron chinensis Schneid. (Rutaceae). Studies have confirmed that EMS, especially in combination with other drugs, can significantly enhance therapeutic efficacy while reducing side effects [10–12]. Our previous research in adjuvant-induced arthritis (AA) rat models demonstrated that EMS alleviates pathological changes, modulates cytokine balance, and inhibits synovial hyperplasia and inflammatory cell infiltration [13–16]. Furthermore, emerging evidence suggests that the anti-inflammatory effects of EMS in periodontitis and endometritis models are associated with the modulation of the NF-κB pathway [17, 18]. These findings imply that EMS may ameliorate RA through analogous mechanism.

The rapid development of network pharmacology, which analyzes the complex “compound-target-disease” interaction networks, provides a powerful tool for investigating multi-component medicines like TCM [19, 20]. Its holistic approach aligns well with the fundamental principles of TCM [21], making it particularly suitable for elucidating the active constituents and mechanisms of herbal formulas.

**Fig. 1:**
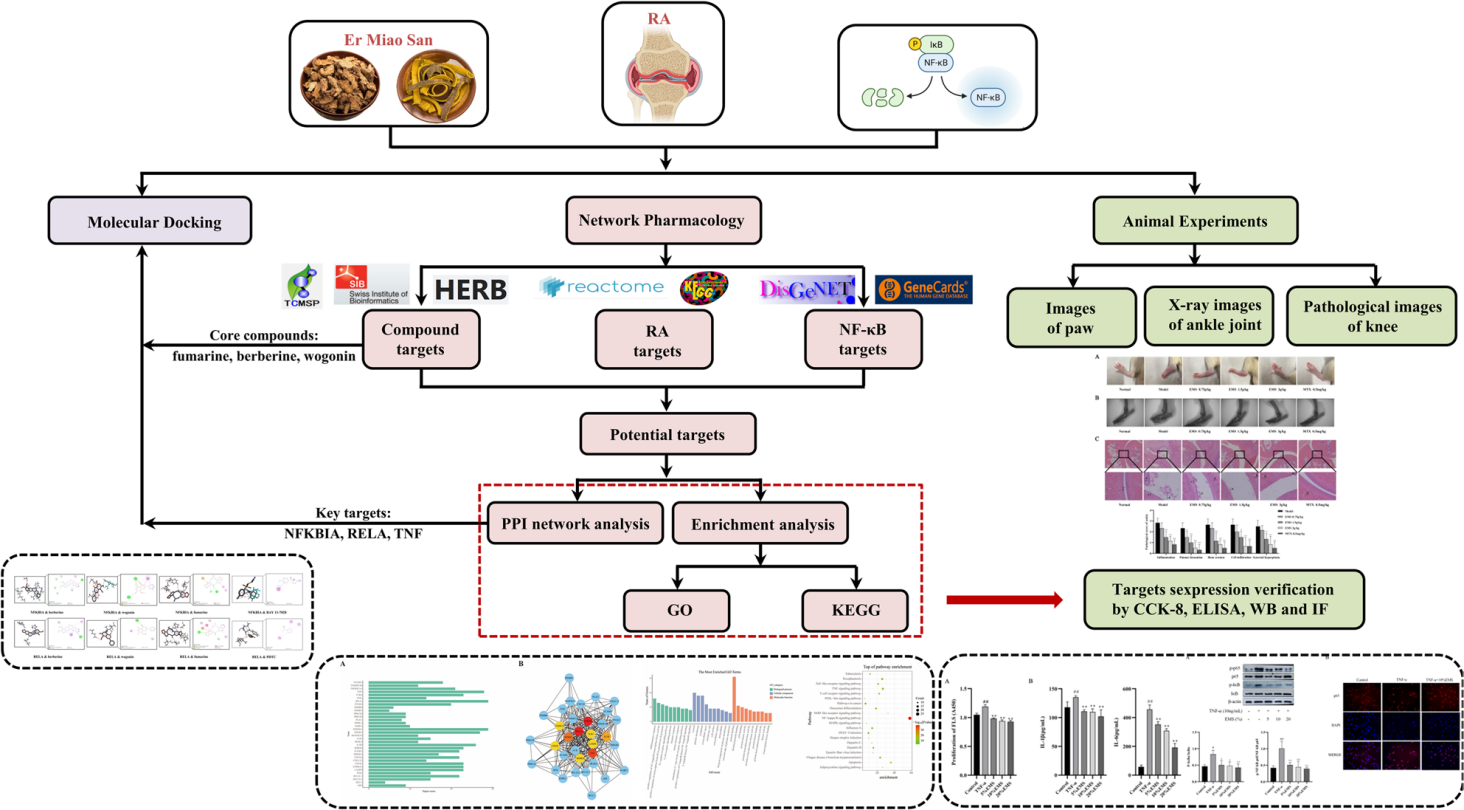
Graphic abstract

In this study, we employed an integrated analysis strategy combining network pharmacology with molecular docking technology to systematically elucidate the intricate relationships among key active components, core targets, and associated biological processes involved in EMS treatment for RA. Molecular docking further validated the binding affinity between key targets and their corresponding active compounds. Finally, we systematically validated the therapeutic effect and mechanism of EMS on RA using a combination of in vivo and in vitro experimental models. The graphic abstract of this study is illustrated in Fig. 1.

## Materials and methods

### EMS chemical ingredients and putative targets

The chemical ingredients of EMS were retrieved from the Traditional Chinese Medicine Systems Pharmacology Database [22] (TCMSP, http://lsp.nwu.edu.cn/tcmsp.php). Active compounds were screened according to the following criteria: drug-likeness (DL) ≥ 0.18 and oral bioavailability (OB) ≥ 30%. Potential target genes of these compounds were predicted using the HERB [23] (HERB, http://herb.ac.cn/) and Swiss Target Prediction [24] (Swiss Target Prediction, http://swisstargetprediction.ch/) databases. HERB provides experimentally documented interactions, while Swiss Target Prediction (for which a probability score > 0 was set as the cutoff) offers computationally predicted targets.

### Collection of RA related targets

RA-associated targets were collected from the GeneCards [25] website (GeneCards, https://www.genecards.org/) and DisGeNET [26] database (DisGeNET, https://www.disgenet.org/home/). The search was performed using “rheumatoid arthritis” as the keyword in both databases. In DisGeNET, the disease concept unique identifier (CUI: C0003873) was also used. Duplicate genes were removed to create a unified set of RA-related targets.

### NF-κB signaling pathway gene acquisition

NF-κB signaling pathway genes were retrieved from the Reactom [27] (Reactom, https://reactome.org/) and Kyoto Encyclopedia of Genes and Genomes [28] (KEGG, https://www.genome.jp/kegg/) databases. Specifically, we extracted genes from the following Reactome pathway IDs (ID: R-HSA-1169091, R-HSA-5676590, R-HSA-5668541, R-HSA-2871837, R-HSA-933542, R-HSA-5607761, R-HSA-5676594, R-HSA-933543, R-HSA-193639, R-HSA-209560) genes and the KEGG pathway map0406.

### RA and intersection target gene enrichment analysis

DAVID [29, 30] (DAVID, https://david.ncifcrf.gov/home.) is a biological information database and a free online analysis tool. It can provide comprehensive biological function annotation information for large-scale gene or protein lists and help users extract biological information from this database. Gene Ontology (GO) functional enrichment analysis, including biological process (BP), cellular composition (CC) and molecular function (MF), describes important information about the target genes. Pathway enrichment analysis indicates significant signaling pathways by classifying the known genome annotation information. The R package Venn Diagram was used to create a Venn diagram for the intersection of EMS-RA-NF-κB signaling pathway target genes. The RA and intersection target genes were separately imported into DAVID (Version 6.8) for GO and pathway enrichment analysis. The identifier was set to “OFFICIAL GENE SYMBOL”, the species was set to “Homo sapiens”, and results with *P* < 0.05 were selected as the restriction. The ggplot2 R package was used to visualize the results.

### Protein-protein interaction network and hub genes

The STRING [31] database (STRING, https://string-db.org/) contains a large number of known and predicted protein-protein interaction (PPI) relationships. The STRING database and Cytoscape (Version 3.6.1) were used to analyze the hub genes and build the network. The intersection target genes were input into the STRING database, the species was set to “Homo sapiens”, and a “confidence score” >0.4 was set as the restriction. The obtained PPI network data were imported into Cytoscape (Version 3.6.1), and the hub genes of intersection target genes were obtained using the plug-in cytoHubba [32, 33] with the degree algorithm. Finally, the intersection of the top 3 hub genes of RA and intersection target genes were taken as the key targets, and a PPI network diagram was constructed for visualization by Cytoscape.

### Network construction

Cytoscape [34]is software that allows for network visualization and analysis. Its core function is to provide a basic functional layout and query network and to form a visual network based on the combination of basic data. Compounds and intersection target genes were transferred into Cytoscape to create a network diagram. Within these graphical networks, the active ingredients and target genes are presented as nodes, whereas the compound-target interactions are expressed as edges.

### Molecular docking

The 3D crystal structures of the key target proteins, NFKBIA (PDB ID: 6Y1J) and RELA (PDB ID: 6NV2 ), were downloaded from the Protein Data Bank [35] (PDB https://www.rcsb.org/) database. Structures were selected based on being from *Homo sapiens*, having high resolution, and being determined by X-ray crystallography. The 3D structures of the active compounds (berberine, wogonin, and fumarine) were downloaded in mol2 format from the TCMSP database and prepared using Chem Office. Molecular docking was performed, and the results were visualized in 2D and 3D using PyMOL 2.4.1 and Discovery Studio 4.5 Client [36, 37].

### Preparation of the EMS

EMS consists of the following two herbs: Atractylodis Rhizoma (Cangzhu, specimen number 1902120322), the rhizome of *Atractylodes lancea* (Thunb.) DC. (Compositae), and Phellodendri Cortex (Huangbai, specimen number 1901200062), the bark of *Phellodendron chinensis* Schneid. (Rutaceae). They were acquired from Anhui Puren Herbal Pieces Co., Ltd (Bozhou, Anhui Province, China) in January 2019, and identified by Dr. Liu SJ (School of Pharmacy, Anhui University of Chinese Medicine). One specimen of each (ID: EMS-19-01) was preserved in the Herbarium of Pharmacy, School of Pharmacy, Anhui University of Traditional Chinese Medicine (Hefei, China).

Following drying and crushing, the herbal mixture (Atractylodis Rhizoma and Phellodendri Cortex) was subjected to aqueous extraction three times (1.5, 1, and 0.5 h for each extraction, respectively). The combined aqueous extract was concentrated to 500–600 mL at 60 °C. The concentrate was then sequentially partitioned five times with equal volumes of petroleum ether and ethyl acetate. The ethyl acetate fraction was collected and concentrated to yield the final EMS extract, which was prepared at doses of 0.3, 0.15, and 0.075 g/mL (based on crude drug weight) for subsequent experiments [14].

### Induction of the AA model and treatment

All animal experiments were performed at the School of Pharmacy, Anhui University of Traditional Chinese Medicine (Hefei, China). The experimental protocol was approved by the Anhui University of Chinese Medicine of Experimental Animal Ethics Committee and adhered to the guidelines for animal care and use (AHUCM-rats-2020016). Male SD rats weighing 160–180 g were acquired from the Animal Department of Anhui Medical University, China, and housed in standard laboratory conditions (under a controlled temperature of 22–26 °C with a 12 h light and 12 h dark period). All rats underwent a 7-day acclimatization period before experimentation. The AA model was induced as previously described [38]. Briefly, complete Freund’s adjuvant (CFA, 10 mg/mL) was prepared by suspending heat-killed Mycobacterium butyricum in sterile liquid paraffin. Model rats received a 100 µL subcutaneous injection of CFA into the left hind metatarsal footpad, while control rats received an equal volume of saline. Successful model induction was confirmed around day 17 by the appearance of redness, swelling, nodules, and limited movement in the limbs, ears, and tail.

Some of the successful AA model rats were used in pharmacodynamic experiments. On the 15th day after immunization, the rats were randomly divided into normal group, AA model group, EMS (3 g/kg, 1.5 g/kg, 0.75 g/kg) group and MTX (0.5 mg/kg) group. EMS was administered by gavage once a day for 14 days and MTX was administered by gavage once every three days for five times. Meanwhile, rats in the normal and AA model groups were gavaged with the same volume of sodium carboxymethylcellulose solution (10 mL/kg).

### Isolation and culture of FLSs

Some successful AA model rats were anesthetized by intraperitoneal injection of sodium pentobarbital at a dose of 30 mg/kg after 14 days of regular feeding and soaked in the newly prepared bromogeramine solution, and synovial tissue was extracted on a super clean table. Based on the methods of isolation and culture of rat FLSs described in the literature [39], the foreign bodies around the tissue blocks were cut off and washed twice with PBS containing 1% penicillin-streptomycin-gentamicin solution (Cat. No. C0223-100 mL, Beyotime, China) in a petri dish and then transferred to Dulbecco’s modified Eagle’s medium (DMEM, Biological Industries, USA) with high glucose supplemented with 20% fetal bovine serum (FBS, Servicebio, China), which was cut into tissue fragments of approximately 1 mm^2^ using ophthalmic scissors. A small volume of medium was added to keep the tissue moist, and the flasks were inverted and placed in a 37 °C, 5% CO₂ incubator (Thermo Scientific, USA) for 4 h to allow tissue adherence. After this period, the flasks were carefully turned right-side up, and complete culture medium was added without disturbing the attached tissue explants. The medium was changed regularly until FLSs migrated from the explants and reached 80–90% confluence. Cells were then passaged using trypsin-EDTA (Cat. No. C0201-100 mL, Beyotime, China) and FLSs from passages 3–8 were used for experiments.

FLSs were cultured in DMEM supplemented with 20% FBS and 1% penicillin/streptomycin. All cells were cultured in a carbon dioxide incubator as described previously. FLSs at passages 3–8 were used for experiments. After three generations of FLSs culture, the shape of FLSs were triangular or fusiform, and immunofluorescence staining showed vimentin^+^, as shown in Supplementary Fig. S1.

### Medicated serum preparation and cell intervention

Normal SD rats were randomly divided into a blank control group and an EMS group (3 g/kg) (calculated using the crude drug), and they were administered intragastrically for 7 days (once a day). One hour after the final administration, rats were anesthetized by intraperitoneal injection of sodium pentobarbital (30 mg/kg) in advance. Blood was collected from the abdominal aorta and centrifuged to isolate the serum. The serum was then inactivated at 56 °C for 1 h, filtered through a 0.22 μm membrane, and stored at -20 °C as the medicated serum.

FLSs were treated with 10 ng/mL TNF-α (Cat. No. 400 − 14, PeproTech, USA) to establish an inflammatory model of RA. FLSs were treated with different concentrations of EMS (5%, 10%, 20%). The experiments were divided into the following groups: Control, TNF-α, TNF-α + 5%EMS, TNF-α + 10%EMS and TNF-α + 20%EMS.

### Ankle x-ray imaging and histopathology

Referring to the previously reported method [40], on the 30th day after immunization, rats were anesthetized by intraperitoneal injection of sodium pentobarbital (30 mg/kg), the left posterior ankle joints of rats were collected and placed in a small animal X-ray irradiator. The imaging parameters were set as follows: resolution 15 LP/mm, voltage 22.0 kV, current 4 mA, and dose 0.1 mGy.

Then, the excess tissues in the ankle joints were removed, fixed with 4% paraformaldehyde solution, decalcified with EDTA decalcification solution, embedded in paraffin, sliced and stained with hematoxylin-eosin (HE). The pathological changes of ankle joint of rats in each group were observed under microscope. The inflammation, pannus formation, bone erosion, cell infiltration and synovial hyperplasia were classified into 4 grades according to the degree of severity: grade 0, no significant changes; grade 1, mild; grade 2, moderate; and grade 3, severe.

### FLS proliferation assays

FLS proliferation was assessed using a Cell Counting Kit-8 (CCK-8, Cat. No. BS350B, Biosharp, China). Cells were seeded in 96-well plates (5 × 10³ cells/well) and treated according to the experimental groupings. After the treatment period, 10 µL of CCK-8 reagent was added to each well, and the plates were incubated for 2 h. The absorbance at 450 nm was measured using a Multiskan Spectrum (Thermo Scientific, USA).

### Enzyme-linked immunosorbent assay (ELISA)

The cell supernatant was collected after culturing for 48 h. An ELISA double antibody sandwich method was used, and the specific operation steps were carried out according to IL-1β (Cat. No. EK301B/3–96, Multisciences Co., Ltd.) and IL-6 (Cat. No. EK306/3–96, Multisciences Co., Ltd.). The OD value of each well read by the enzyme labeling instrument was taken as the ordinate, the concentration of the standard was taken as the abscissa, and the curve was drawn. The corresponding concentration range was found according to the OD value of the sample.

### Western blot

Cells were lysed on ice using RIPA lysis buffer supplemented with phenylmethanesulfonyl fluoride (PMSF, Cat. No. ST506, Beyotime, China) and phosphatase inhibitors (Cat. No. P1081, Beyotime, China). The lysates were collected and subjected to three freeze-thaw cycles at -20 °C, followed by centrifugation at 14,000 rpm for 15 min to remove cellular debris. Protein concentration was determined with a BCA Protein Assay Kit. Equal amounts of total protein were separated by sodium dodecyl sulfate-polyacrylamide gel electrophoresis (SDS-PAGE) and transferred onto a polyvinylidene fluoride (PVDF) membrane. After blocking with 5% non-fat milk, the membranes were incubated overnight at 4 °C with primary antibodies against IκBα (Cat. No. 4814, 1:1000, Cell Signaling Technology, USA), p-IκBα (Cat. No. 2859, 1:1000, Cell Signaling Technology, USA), NF-κB p65 (Cat. No. 8242, 1:1000, Cell Signaling Technology, USA) and p-NF-κB p65 (Cat. No. 3033, 1:1000, Cell Signaling Technology, USA. Subsequently, the membranes were washed with Tris-buffered saline containing Tween 20 (TBST) and incubated with appropriate secondary antibodies for 2 h at room temperature. Protein bands were visualized using an enhanced chemiluminescence (ECL) reagent and imaged with the Amersham Imager 600 system (GE Healthcare Bio-Sciences AB, USA). Band intensities were quantified using ImageJ software.

### Immunofluorescence

Cells on 24-well plates were washed with PBS and fixed in 4% paraformaldehyde (Cat. No. BL539A, Biosharp, China) for 30 min. After three washes with PBS, the cells were blocked using Immunol Staining Blocking Buffer (Cat. No. P0102, Beyotime, China) for 1 h at room temperature. The cells were then incubated with primary antibodies overnight at 4 °C, followed by three 5-min washes with PBS. Subsequently, the cells were incubated with secondary antibodies (Cat. No. SA00013-4, Proteintech, USA) for 1 h at room temperature in the dark. Following two additional 5-min washes, the cells were counterstained with DAPI (Cat. No. C1005, Beyotime, China) for 5 min in the dark and finally examined under an immunofluorescence microscope (Leica, Germany).

### Statistical analysis

Data are presented as the mean ± standard deviation (SD). The differences between groups were analyzed by one-way analysis of variance (ANOVA) using Prism 8 software (GraphPad Software, USA). A *P* value < 0.05 indicated statistical significance.

## Results

### Screening of active components and targets in EMS, RA and NF-κB signaling pathway

To identify the active components of EMS with potential efficacy against RA, we retrieved 46 candidate compounds from the TCMSP database: 37 from *Phellodendri Cortex* and 9 from *Atractylodis Rhizoma.* These compounds were screened based on their OB and DL properties, all of which met the drug-likeness criteria (OB ≥ 30% and DL ≥ 0.18) and were considered candidate compounds for subsequent analysis (see Supplementary Table S1).

**Fig. 2:**
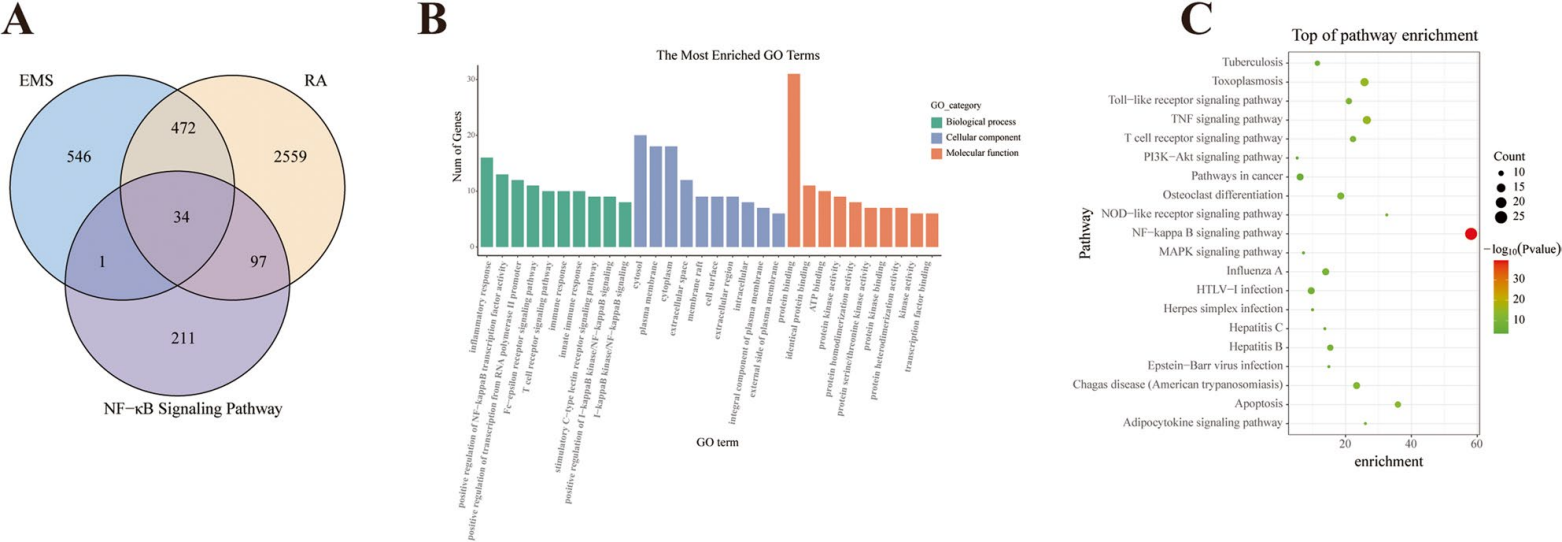
Enrichment analysis. **A** Venn diagram identifies targets common to EMS, RA, and the NF-κB pathway. **B** GO and (**C**) KEGG pathway enrichment analyses of the overlapping targets

The potential targets of these active ingredients were predicted using the HERB and Swiss Target Prediction databases, yielding 1053 targets. Meanwhile, 3162 RA-related targets were collected from the GeneCards and DisGeNET databases, and 343 genes associated with the NF-κB signaling pathway were sourced from KEGG and Reactome. A Venn diagram generated with the Venn Diagram R package revealed 34 common targets at the intersection among EMS, RA and the NF-κB pathway (Fig. 2A). These overlapping targets suggest a potential mechanism by which EMS may exert its anti- RA effects through modulation of the NF-κB signaling pathway.

### Enrichment analysis of intersection targets

The 34 intersection targets were further analyzed through GO and pathway enrichment analyses using the DAVID database. The bar plot of the top 10 significantly enriched GO terms (Fig. 2B) showed that the BP was primarily associated with inflammatory response and immune regulation, including ‘inflammatory response’, ‘positive regulation of NF-κB transcription factor activity’, and ‘Fc-epsilon receptor signaling pathway’. The CC included the ‘cytosol’, ‘plasma membrane’, and ‘extracellular space’. Regarding MF, the most significant terms were ‘protein binding’ and ‘ATP binding’. Pathway analysis (Fig. 2C) further indicated associations with the ‘TNF signaling pathway’ and ‘osteoclast differentiation’. Collectively, these enrichment results underscore the central role of the NF-κB signaling pathway in the mechanism of EMS, suggesting that it may coordinate other critical processes in RA—such as TNF-mediated inflammation and osteoclast differentiation—to drive disease pathogenesis.

### Construction of the compound-target and protein-protein interaction networks

**Fig. 3:**
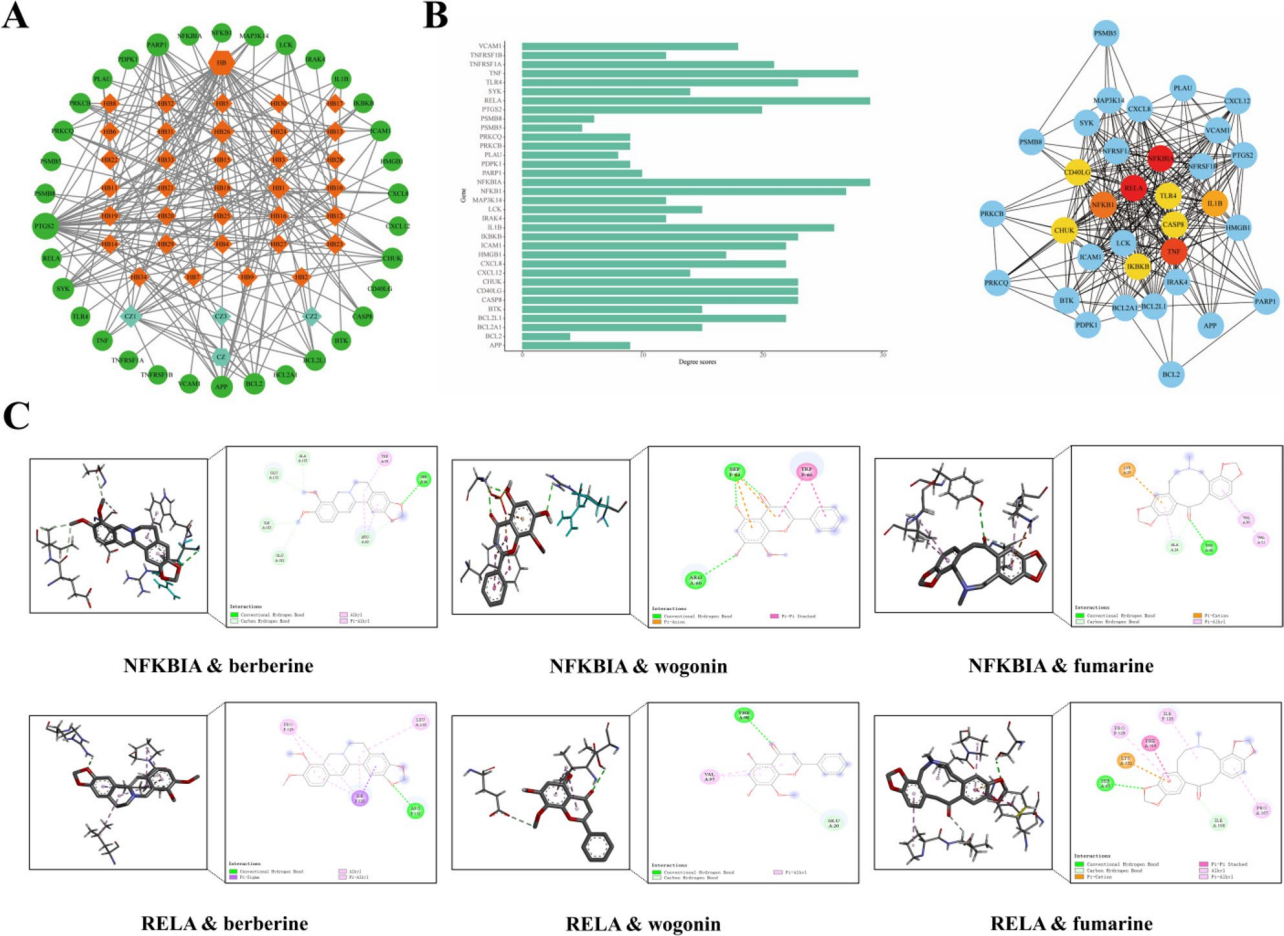
Network analysis and molecular docking. **A** Network of “drug - active ingredient - target”. **B** Hub targets identified from the PPI network. **C** Molecular docking diagram of core components and targets

The compound-target network was constructed to visualize the interactions between the 34 intersection targets and their corresponding active compounds from EMS (Fig. 3A). Node connectivity analysis, based on degree values, identified berberine, wogonin, and fumarine as the most highly connected compounds, suggesting their potential role as key mediators of the therapeutic effects of EMS against RA via network regulation.

To further investigate the protein-level interactions among the 34 intersection targets, a protein-protein interaction (PPI) network was established using the STRING database and visualized with Cytoscape (Fig. 3B). This network consisted of 34 nodes and 287 edges. Ranking the nodes by degree (Supplementary Table S2) highlighted NFKBIA (degree = 29; encodes IκBα), RELA (degree = 29; p65), and TNF (degree = 28) as the most interconnected proteins, suggesting their central roles in the network and nominating them as candidate key therapeutic targets for EMS.

### Molecular docking validation

To validate the interactions between the key EMS compounds and the core targets, molecular docking was performed using AutoDock Vina. The binding energies of berberine, wogonin, and fumarine to NFKBIA and RELA were calculated and compared to those of known inhibitors (BAY 11-7082 for NFKBIA and JSH-23 for RELA, which exhibited binding energies of -6.4 kcal/mol and − 6.9 kcal/mol, respectively). All three compounds exhibited more negative binding energies than the reference inhibitors: berberine (-8.2 and − 7.3 kcal/mol), wogonin (-7.2 and − 7.0 kcal/mol), and fumarine (-8.9 and − 8.7 kcal/mol) for NFKBIA and RELA, respectively. Furthermore, visualization of the optimal docking poses confirmed that each compound forms conventional hydrogen bonds with the respective targets (Fig. 3C). These results indicate a high binding affinity and support the hypothesis that these compounds play a pivotal role in the mechanism by which EMS treats RA.

### Effect of EMS on pathological changes of ankle joint in AA rats

**Fig. 4:**
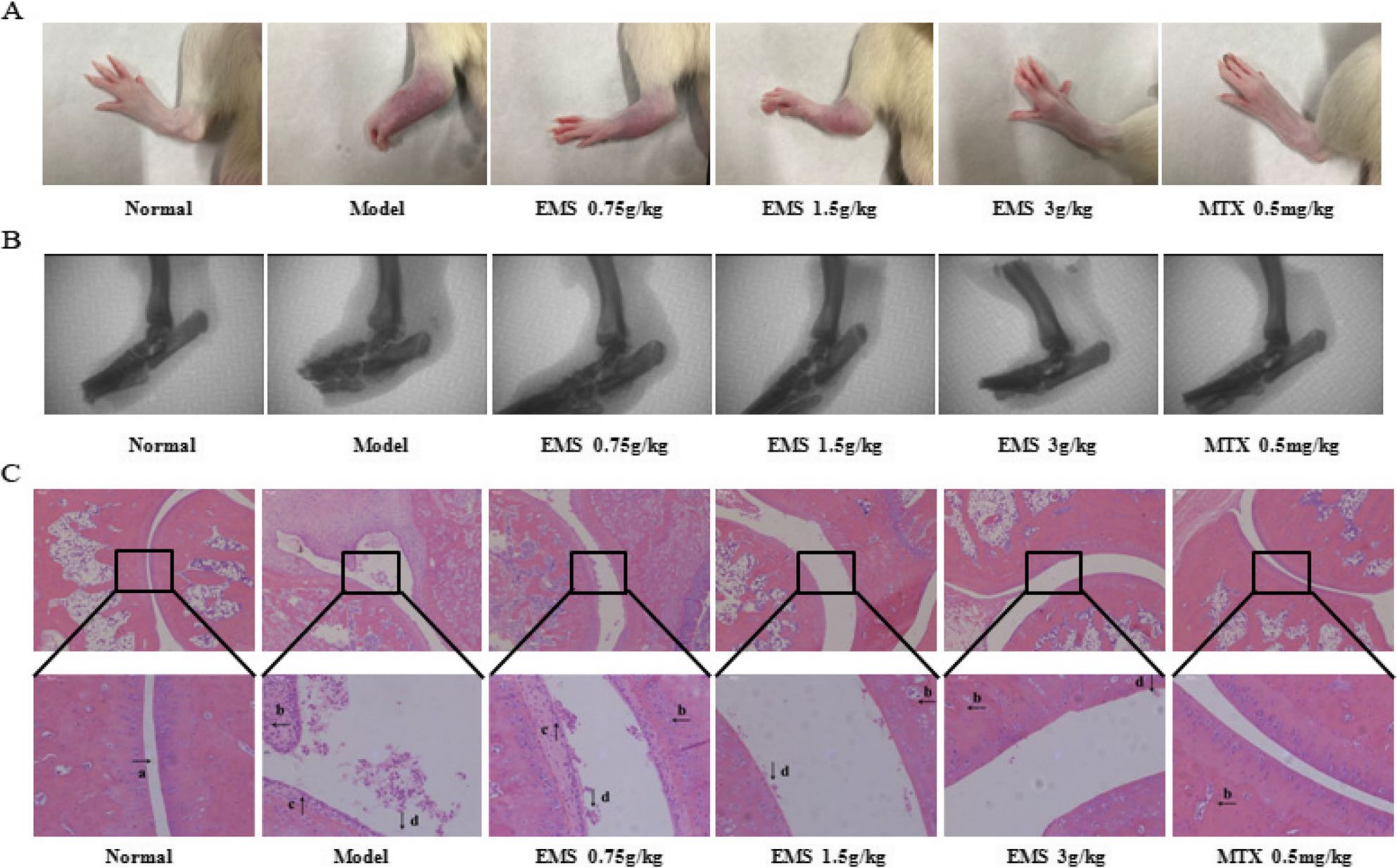
Effect of EMS-Ethyl Acetate Extract on Ankle Joint Pathology in AA Rats ($\:\stackrel{-}{x}$±*s*, *n* = 6). **A** The image of paws of AA rats (day 30). **B** X-ray images of ankle joints. **C** Histopathological images of ankle joints; a: gristle; b: lymphocyte infiltration; c: synovial tissue hyperplasia; d: pannus formation. ^*^P < 0.05, ^**^P < 0.01, vs. the model group

To evaluate the anti-arthritic efficacy of EMS, we assessed its effects on ankle joint pathology in AA rats. As shown in Fig. 4A, model rats exhibited severe paw swelling and multiple articular nodules. Treatment with EMS or MTX significantly alleviated this swelling compared to the model group. X-ray analysis (Fig. 4B) revealed obvious soft tissue swelling, joint deformation, and bone erosion in the model group, all of which were markedly improved in the treatment groups, with joints exhibiting greater structural integrity. Histopathological assessment of HE staining sections (Fig. 4C) showed substantial inflammatory cell infiltration, synovial hyperplasia, pannus formation, and cartilage erosion in the model group. These pathological changes were substantially reduced by EMS treatment, resulting in significantly lower ankle joint severity scores in both the EMS and MTX groups. Collectively, these results demonstrate that EMS ameliorates ankle joint pathology and exerts a protective effect in AA rats (Table 1).

**Table 1:**
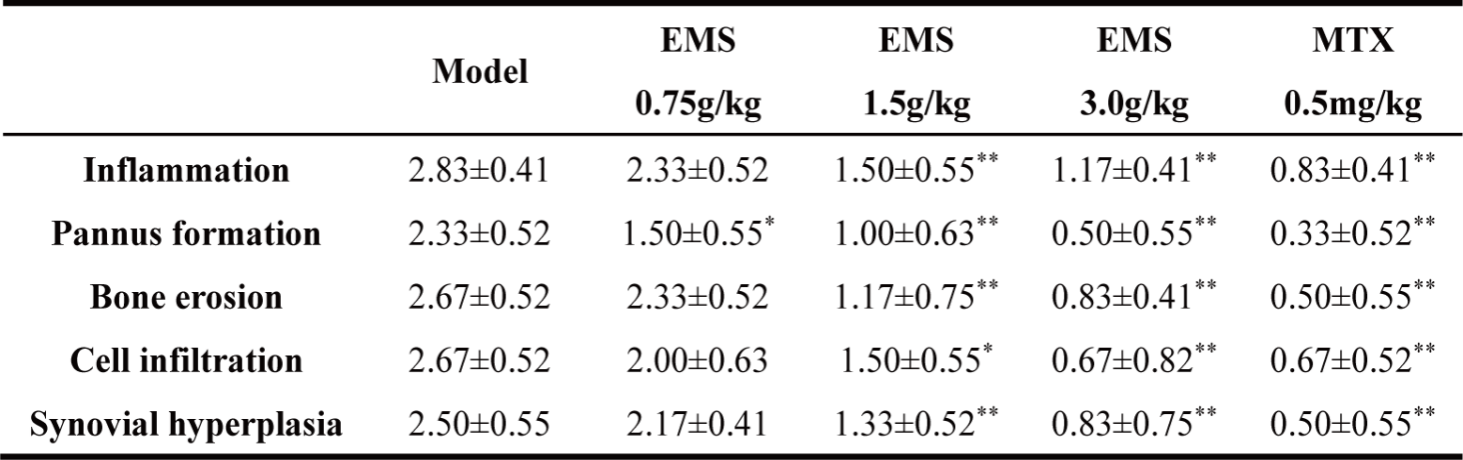
Effect of EMS on pathological changes of ankle joint in AA rats

^*^P < 0.05, ^**^P < 0.01, vs. the model group ($\:\stackrel{-}{x}$±*s*, *n* = 6)

### EMS inhibited FLS proliferation and inflammatory cytokine expression

**Fig. 5:**
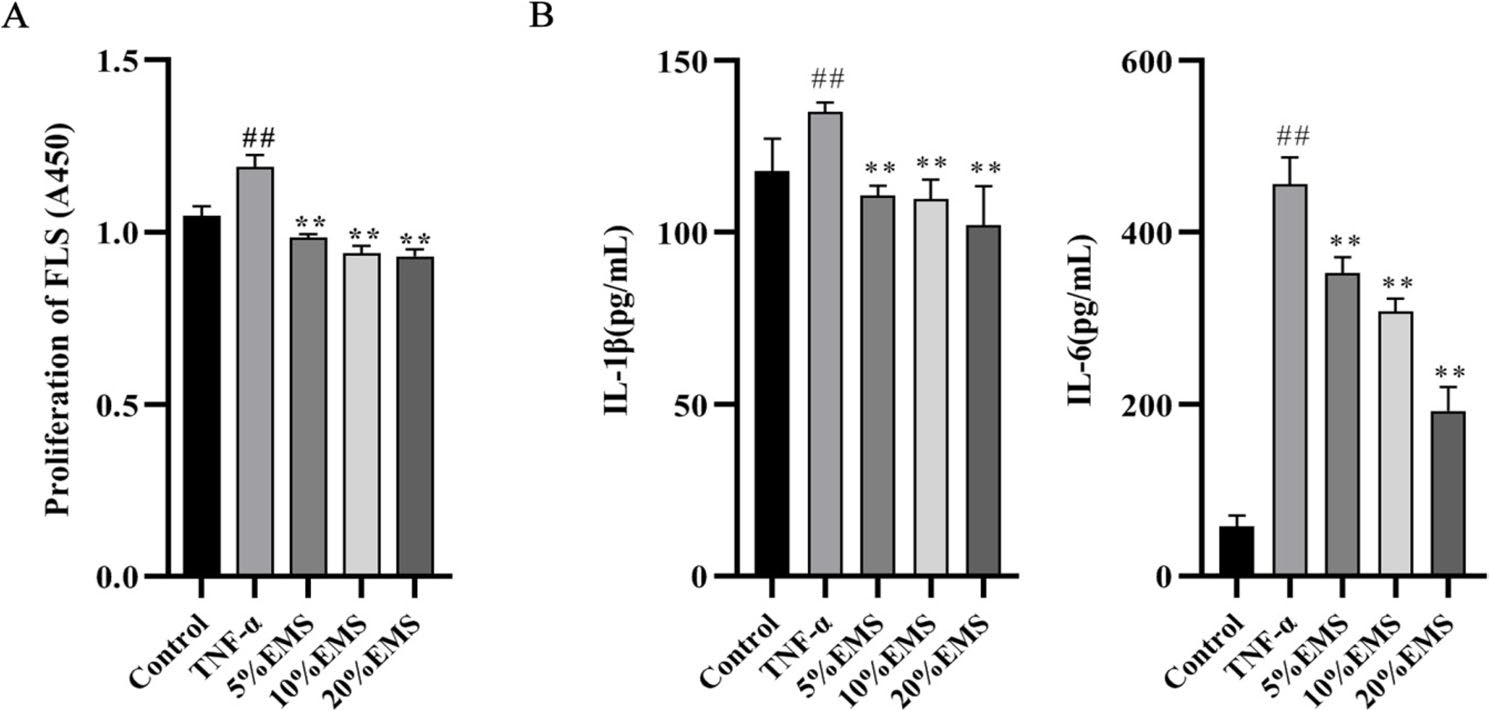
EMS reduced FLS proliferation and inflammatory cytokine expression in a concentration-dependent manner. **A** EMS reduced FLS proliferation. **B** EMS inhibited IL-1β and IL-6 expression in TNF-α-treated FLS supernatant. Data are shown as the means ± SDs of six independent experiments. ^##^*P* < 0.01, vs. the control group; **P < 0.01, vs. the TNF-α group

FLSs, key effector and target cells in RA, exhibit abnormal proliferation that drives disease pathology [41]. We therefore evaluated the effect of EMS on this process. As shown in Fig. 5A, TNF-α stimulation significantly promoted FLS proliferation compared with the control, whereas co-treatment with EMS (5%, 10%, and 20%) markedly suppressed this abnormal growth in a concentration-dependent manner. We next investigated the anti-inflammatory effect of EMS. ELISA results indicated that TNF-α stimulation significantly upregulated the secretion of IL-1β and IL-6, which were substantially attenuated by EMS treatment (Fig. 5B). Collectively, these results demonstrate that EMS effectively inhibits the abnormal proliferation of RA-FLSs and the overproduction of inflammatory cytokines.

### EMS inhibited the phosphorylation of NF-κB p65, IκBα protein expression and p65 nuclear translocation in TNF-α-induced FLSs

**Fig. 6:**
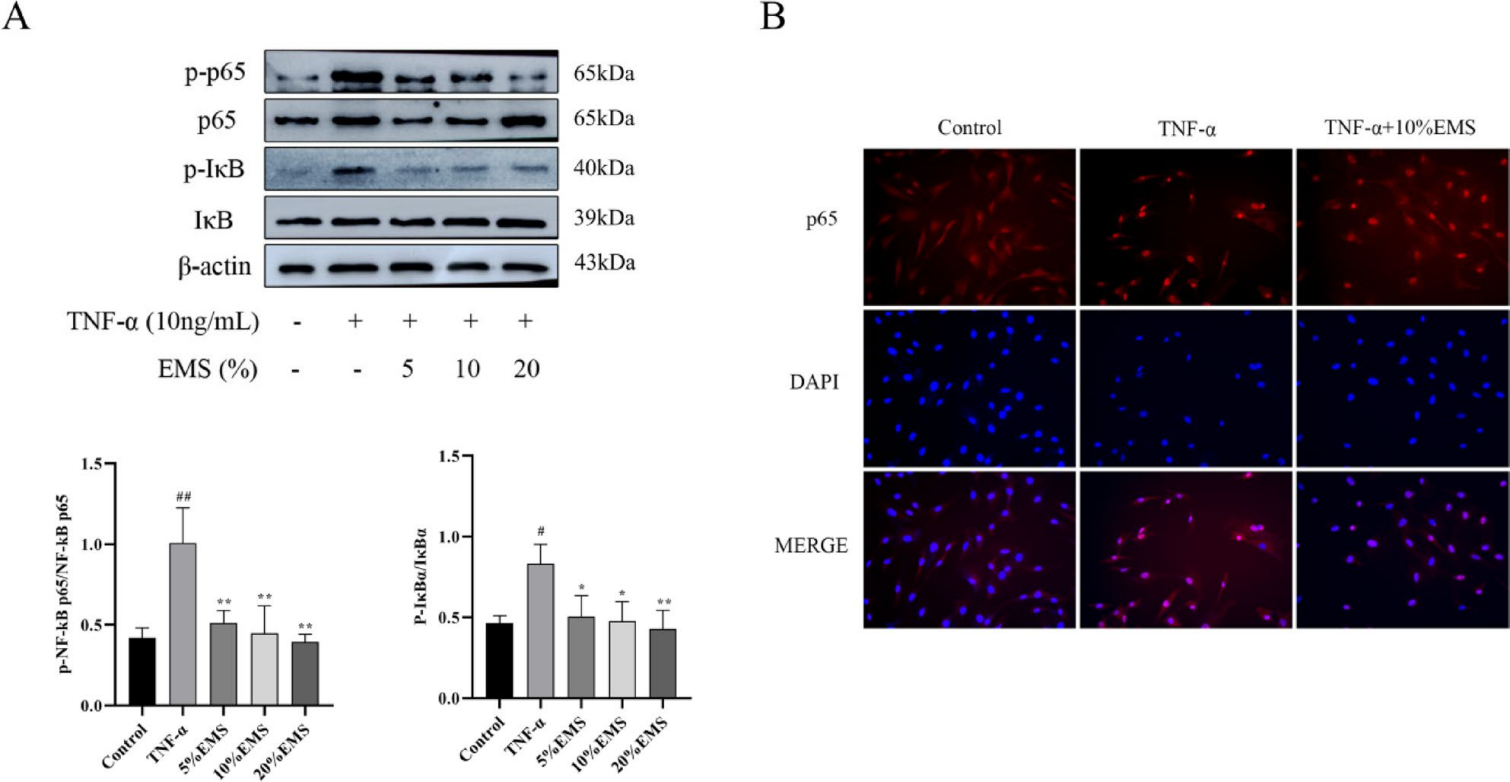
EMS inhibits TNF-α-induced NF-κB signaling in FLS. **A** EMS reduced the phosphorylation of IκBα and p65. **B** EMS (10%) attenuated p65 nuclear translocation (200×; scale bars = 20 μm). Data are shown as the means ± SDs of three independent experiments. ^*#*^*P* < 0.05, ^*##*^*P* < 0.01, vs. the control group; ^*^P < 0.05, ^**^P < 0.01, vs. the TNF-α group

To further validate the network pharmacology predictions, we investigated the effect of EMS on the NF-κB pathway in FLSs. Western blot analysis demonstrated that EMS treatment significantly inhibited TNF-α-induced phosphorylation of IκBα and NF-κB p65 (Fig. 6A). Furthermore, immunofluorescence staining was used to evaluate the subcellular localization of NF-κB p65. As shown in Fig. 6B, TNF-α stimulation induced a pronounced nuclear translocation of NF-κB p65, which was markedly inhibited by co-treatment with EMS (10%), resulting in significantly less p65 within the nucleus. Collectively, these results demonstrate that the anti-inflammatory effect of EMS is mediated, at least in part, through suppression of the NF-κB signaling pathway.

## Discussion

RA is a multifaceted disease driven by complex genetic and biological pathways [42]. FLSs play a central role in RA progression by releasing inflammatory cytokines and other mediators, which contribute to synovial hyperplasia and eventual joint destruction [43]. Enhanced NF-κB activity in the inflammatory tissues of RA patients underscores its substantial role in perpetuating the disease process [44]. This pathway’s activation exacerbates chronic inflammation, fostering a cycle of tissue damage and functional impairment within the affected joints. Understanding these intricate mechanisms is crucial for developing targeted therapies aimed at mitigating inflammation and preserving joint function in RA patients. Currently, the primary objective of RA treatment is to alleviate or substantially decrease disease activity to prevent joint injury or disability [45]. Disease-modifying antirheumatic drugs (DMARDs) can suppress overactive immune function and inflammation in RA patients, although their use may lead to various adverse effects [46, 47]. The traditional herbal formula EMS has demonstrated therapeutic potential against RA, but its systematic mechanism of action remains incompletely understood.

In this study, network pharmacology analyses identified berberine, wogonin, and fumarine as key candidate compounds in EMS, with NFKBIA, RELA, and TNF emerging as core targets. These findings are consistent with the ‘multi-component, multi-target’ characteristic of EMS, supporting the holistic philosophy of TCM formulas. Notably, the three key compounds were predicted to concurrently engage pivotal nodes of the NF-κB pathway, suggesting a potential synergistic mechanism where EMS may simultaneously stabilize the cytoplasmic NF-κB complex through IκBα (encoded by NFKBIA) while inhibiting the nuclear activity of the transcription factor p65 (RELA). This coordinated action suggests a theoretical basis for achieving a broader anti-inflammatory effect than targeting a single component, a hypothesis that merits experimental verification. Molecular docking further supported this hypothesis, predicting that berberine, wogonin, and fumarine exhibit theoretically stronger binding affinities to NFKBIA and RELA than known reference inhibitors [48].

While our integrated approach successfully identified key candidate compounds and targets, it is imperative to acknowledge the inherent speculative nature of such predictive methodologies. Computational analyses can identify candidates but cannot confirm biological activity or therapeutic relevance [49, 50]. Therefore, to bridge this gap, we subsequently validated these predictions through a series of in vivo and in vitro experiments. In an AA rat model, EMS treatment effectively alleviated ankle joint swelling, deformation, synovial hyperplasia and bone erosion, confirming its therapeutic potential at the organismal level. Given the pivotal role of FLS in RA pathogenesis, we further investigated the cellular effects of EMS. The results demonstrated that EMS concentration-dependently suppressed TNF-α-induced FLS proliferation and reduced the secretion of the pro-inflammatory cytokines IL-1β and IL-6.

We then focused on the NF-κB pathway, a key prediction from our network analysis. NF-κB activation depends on IκBα phosphorylation and subsequent p65 nuclear translocation, which triggers pro-inflammatory gene expression [51–53]. Western blot and immunofluorescence analyses unequivocally demonstrated that EMS inhibits the phosphorylation of IκBα and p65, thereby preventing p65 nuclear translocation in TNF-α-induced FLS. Collectively, these results provide direct mechanistic evidence linking the anti-inflammatory effect of EMS to the suppression of the NF-κB pathway in FLS, which is consistent with the reported activities of its key compounds, such as berberine’s known inhibition of p65 nuclear translocation [54–56].

From a translational medicine perspective, the “multi-component, multi-target” nature of EMS is central to decoding its traditional efficacy and building a modern evidence base. Unlike biologics or conventional DMARDs, EMS appears capable of simultaneously modulating multiple key nodes within the NF-κB signaling pathway. Our integrated analysis suggests a concurrent engagement of both IκBα and p65, providing a mechanistic basis for its synergistic modulation. This polypharmacological theoretically suggests the potential for a more favorable balance between efficacy and safety compared to single-target approaches, potentially reducing the risk of pathway reactivation. This represents a key scientific question in translating an empirical herbal preparation into a modern botanical drug, though this presumed advantage requires rigorous testing through direct comparative studies with established therapies.

In summary, by integrating computational prediction with experimental validation, this study elucidates that EMS alleviates RA-related inflammation primarily through the suppression of the NF-κB pathway in FLS. Preliminary findings also support a promising safety profile. This work provides critical mechanistic evidence for EMS, taking a key first step in its translation. Moving forward, to achieve its full transition from an empirical preparation to an evidence-based botanical drug, EMS must still address common challenges for complex natural products, including formulation standardization, comprehensive pharmacokinetic studies, and long-term safety evaluation. Ultimately, deciphering the ‘multi-component, multi-target’ synergy of EMS may not only advance its own development but also provide a translational roadmap for modernizing other TCM formulas.

## Conclusion

This study systematically elucidated the anti-arthritic mechanism of EMS using an integrated strategy combining network pharmacology, molecular docking, and experimental validation. We identified berberine, wogonin, and fumarine as potential key active compounds and demonstrated that EMS exerts its therapeutic effects primarily by suppressing the NF-κB pathway in FLSs. This suppression was confirmed by the inhibition of IκBα/p65 phosphorylation and p65 nuclear translocation, and subsequent down-regulation of pro-inflammatory cytokine production. Collectively, this work not only reveals a central mechanism behind the efficacy of EMS but also provides a practical framework for bridging computational prediction and functional validation in TCM research. These findings support the further development of EMS as a multi-target therapy for RA.

## Supplementary Information

Supplementary Material 1.

Supplementary Material 2.

Supplementary Material 3.

Supplementary Material 4.

## Data Availability

The database links used in this article are as follows: TCMSP database (https://old.tcmsp-e.com/tcmsp.php), HERB database (http://herb.ac.cn/), SwissTargetPrediction (http://swisstargetprediction.ch/), GeneCards (https://www.genecards.org/), DisGeNET database (https://www.disgenet.org/home/), Reactom (https://reactome.org/) and KEGG databases (https://www.genome.jp/kegg/), DAVID (https://david.ncifcrf.gov/), STRING database (https://string-db.org/), PDB database(https://www.rcsb.org/). The article and its additional fles include all the data generated or analyzed in this study.

## Abbreviations

AA: Adjuvant-induced arthritis
GO: Gene ontology
BP: Biological process
HE: Hematoxylin-eosin staining
CC: Cellular component
HRP: Horseradish peroxidase
CFA: Complete Freund’s adjuvant
IL-6: Interleukin 6
DL: Drug-likeness
MF: Molecular function
ECL: Enhanced chemiluminescence
OB: Oral bioavailability
ELISA: Enzyme-linked immunosorbent assay
PPI: Protein-protein interaction
EMS: Er Miao San
RA: Rheumatoid arthritis
FLSs: Fibroblast-like synoviocytes
SDS-PAGE: Sodium dodecyl sulfate-polyacrylamide gel electrophoresis
FBS: Fetal bovine serum
TCM: Traditional Chinese medicine
