# Supplementary material for: Exploring the molecular mechanism of Er Miao San for treating rheumatoid arthritis based on network pharmacology
Source: BMC Complement Med Ther. 2026 Apr 10;26:187. doi: 10.1186/s12906-026-05359-6 (PMC13188287; doi:10.1186/s12906-026-05359-6)
Supplement: Supplementary file 1 — Supplementary Material 1. [file 12906_2026_5359_MOESM1_ESM.docx]

**Supplementary Table S1.** **Active components of Er Miao San**

| ID | MOL ID | Name | OB/% | DL |
| --- | --- | --- | --- | --- |
| HB1 | MOL001454 | berberine | 36.86 | 0.78 |
| HB2 | MOL001458 | coptisine | 30.67 | 0.86 |
| HB3 | MOL002636 | Kihadalactone A | 34.21 | 0.82 |
| HB4 | MOL013352 | Obacunone | 43.29 | 0.77 |
| HB5 | MOL002641 | Phellavin_qt | 35.86 | 0.44 |
| HB6 | MOL002643 | delta 7-stigmastenol | 37.42 | 0.75 |
| HB7 | MOL002644 | Phellopterin | 40.19 | 0.28 |
| HB8 | MOL002651 | Dehydrotanshinone II A | 43.76 | 0.40 |
| HB9 | MOL002652 | delta7-Dehydrosophoramine | 54.45 | 0.25 |
| HB10 | MOL002656 | dihydroniloticin | 36.43 | 0.81 |
| HB11 | MOL002659 | kihadanin A | 31.60 | 0.70 |
| HB12 | MOL002660 | niloticin | 41.41 | 0.82 |
| HB13 | MOL002662 | rutaecarpine | 40.30 | 0.60 |
| HB14 | MOL002663 | Skimmianin | 40.14 | 0.20 |
| HB15 | MOL002666 | Chelerythrine | 34.18 | 0.78 |
| HB16 | MOL000449 | Stigmasterol | 43.83 | 0.76 |
| HB17 | MOL002668 | Worenine | 45.83 | 0.87 |
| HB18 | MOL002670 | Cavidine | 35.64 | 0.81 |
| HB19 | MOL002671 | Candletoxin A | 31.81 | 0.69 |
| HB20 | MOL002672 | Hericenone H | 39.00 | 0.63 |
| HB21 | MOL002673 | Hispidone | 36.18 | 0.83 |
| HB22 | MOL000358 | beta-sitosterol | 36.91 | 0.75 |
| HB23 | MOL000622 | Magnograndiolide | 63.71 | 0.19 |
| HB24 | MOL000762 | Palmidin A | 35.36 | 0.65 |
| HB25 | MOL000785 | palmatine | 64.60 | 0.65 |
| HB26 | MOL000787 | Fumarine | 59.26 | 0.83 |
| HB27 | MOL000790 | Isocorypalmine | 35.77 | 0.59 |
| HB28 | MOL000098 | quercetin | 46.43 | 0.28 |
| HB29 | MOL001131 | phellamurin_qt | 56.60 | 0.39 |
| HB30 | MOL001455 | (S)-Canadine | 53.83 | 0.77 |
| HB31 | MOL001771 | poriferast-5-en-3beta-ol | 36.91 | 0.75 |
| HB32 | MOL002894 | berberrubine | 35.74 | 0.73 |
| HB33 | MOL005438 | campesterol | 37.58 | 0.71 |
| HB34 | MOL006392 | dihydroniloticin | 36.43 | 0.82 |
| HB35 | MOL006401 | melianone | 40.53 | 0.78 |
| HB36 | MOL006413 | phellochin | 35.41 | 0.82 |
| HB37 | MOL006422 | thalifendine | 44.41 | 0.73 |
| CZ1 | MOL000173 | wogonin | 30.68 | 0.23 |
| CZ2 | MOL000179 | 2-Hydroxyisoxypropyl-3-hydroxy-7-isopentene-2,3-dihydrobenzofuran-5-carboxylic | 45.20 | 0.20 |
| CZ3 | MOL000184 | NSC63551 | 39.25 | 0.76 |
| CZ4 | MOL000186 | Stigmasterol 3-O-beta-D-glucopyranoside_qt | 43.83 | 0.76 |
| CZ5 | MOL000188 | 3β-acetoxyatractylone | 40.57 | 0.22 |
| CZ6 | MOL000085 | beta-daucosterol_qt | 36.91 | 0.75 |
| CZ7 | MOL000088 | beta-sitosterol 3-O-glucoside_qt | 36.91 | 0.75 |
| CZ8 | MOL000092 | daucosterin_qt | 36.91 | 0.76 |
| CZ9 | MOL000094 | daucosterol_qt | 36.91 | 0.76 |
