# Supplementary figures and images for: Exploring the molecular mechanism of Er Miao San for treating rheumatoid arthritis based on network pharmacology
Source: BMC Complement Med Ther. 2026 Apr 10;26:187. doi: 10.1186/s12906-026-05359-6 (PMC13188287; doi:10.1186/s12906-026-05359-6)

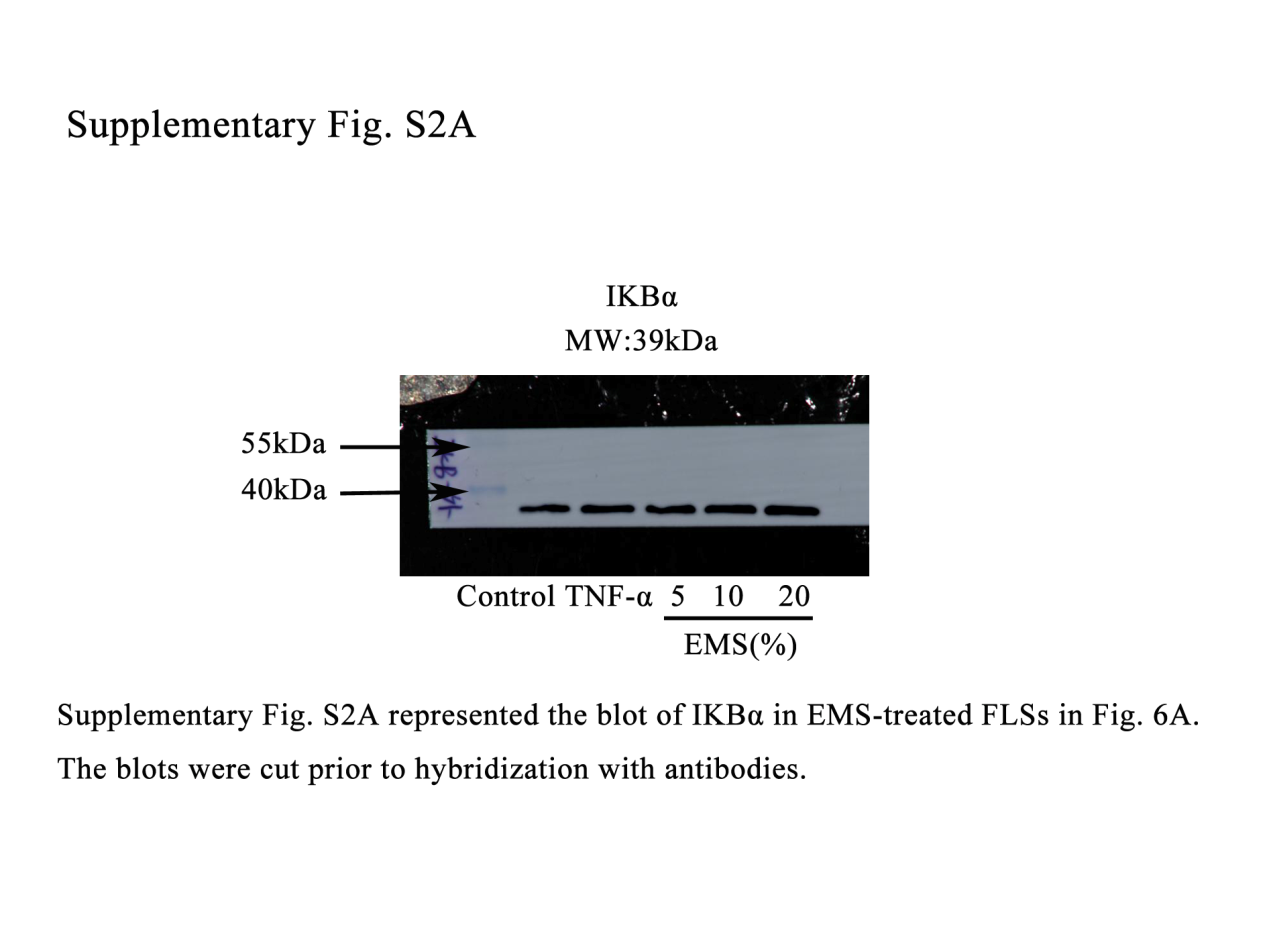


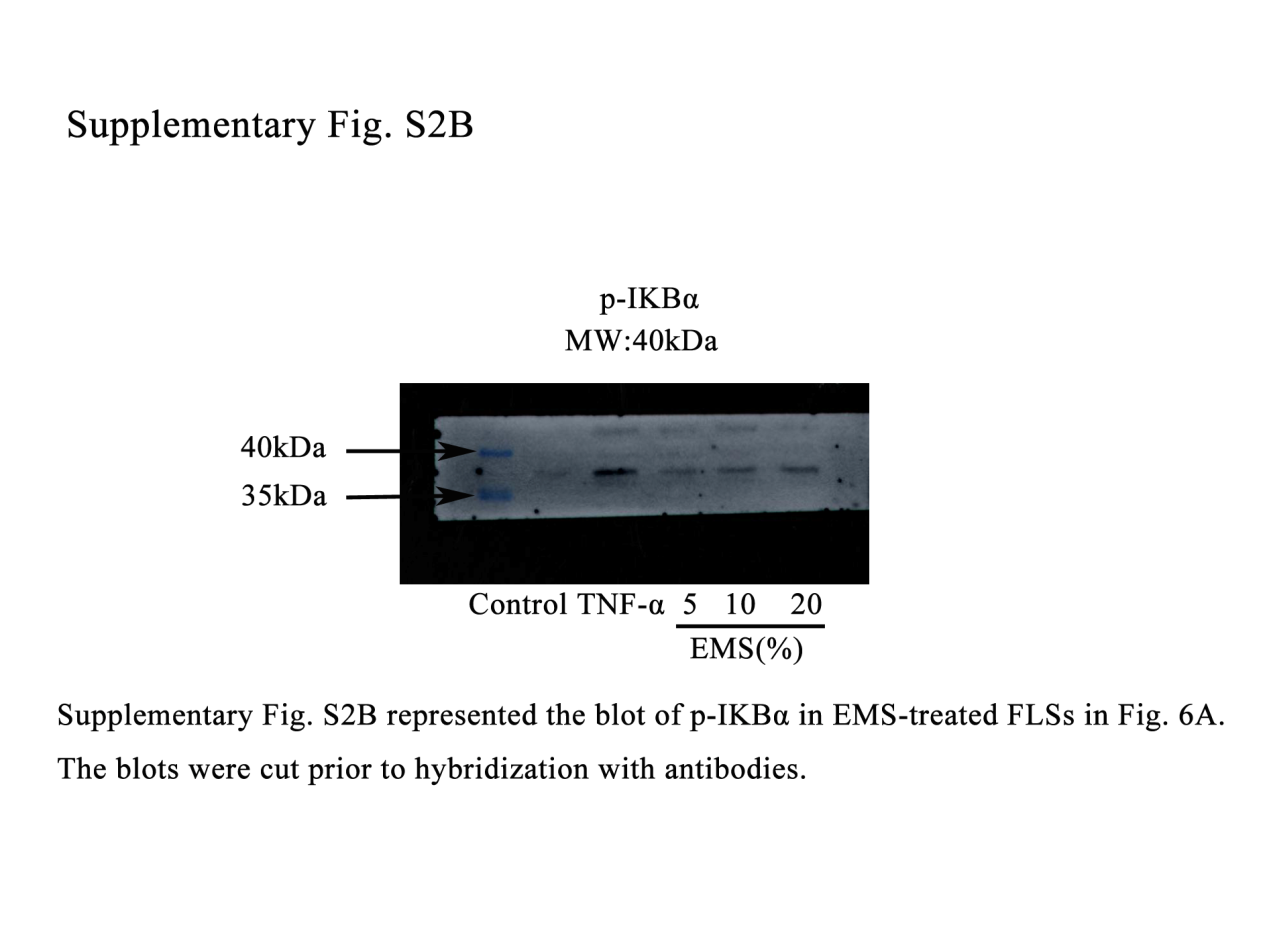


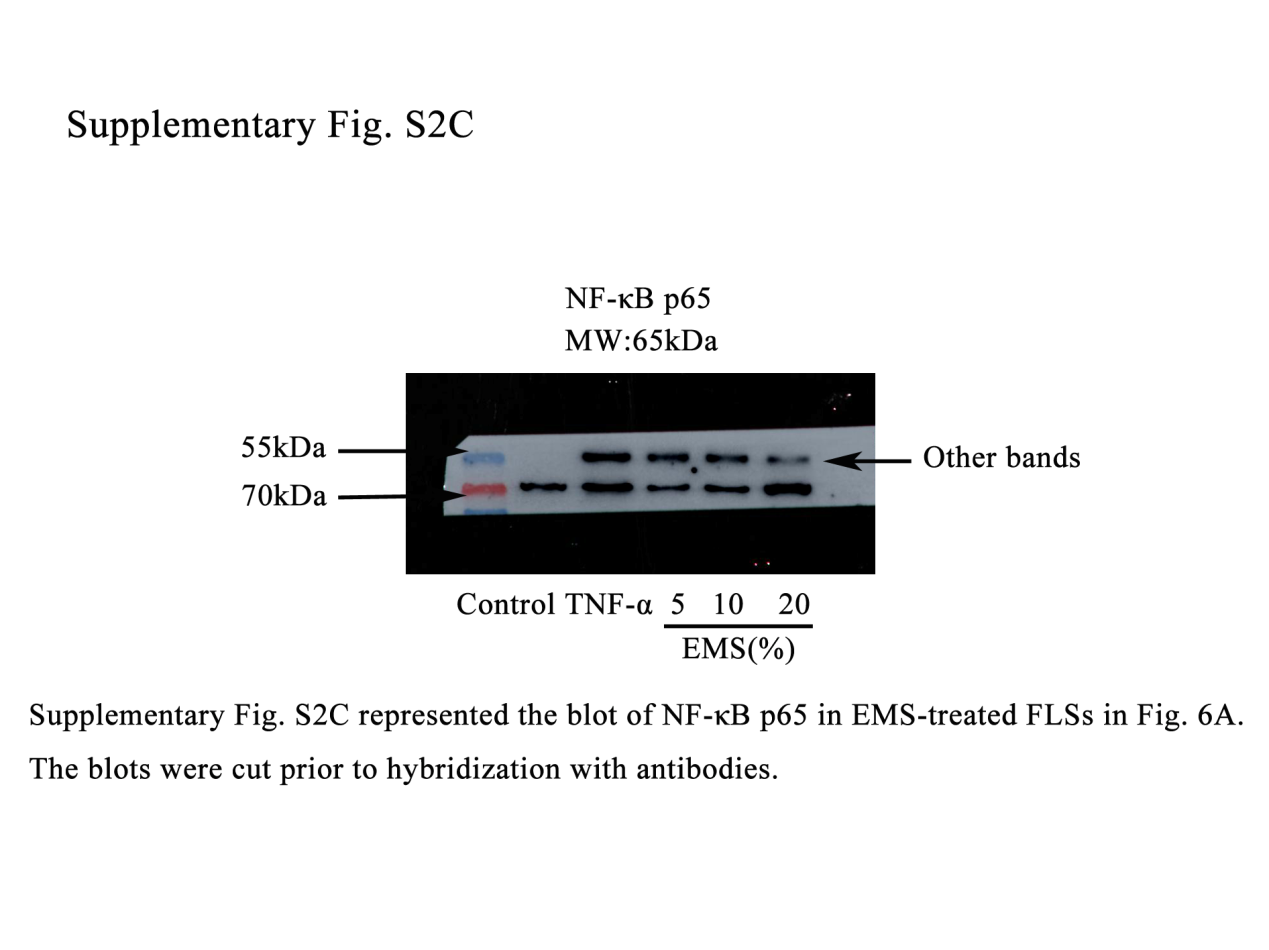


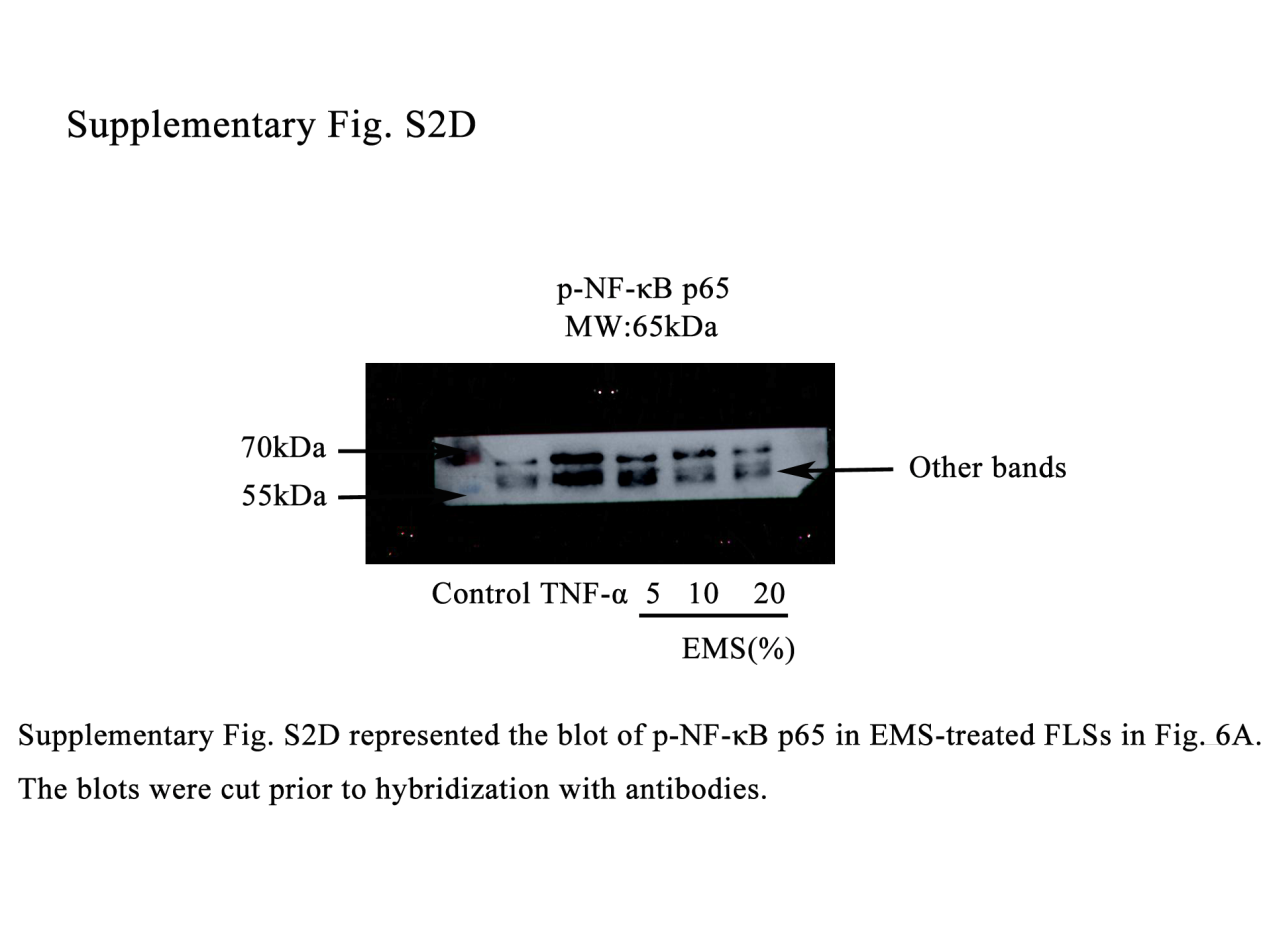


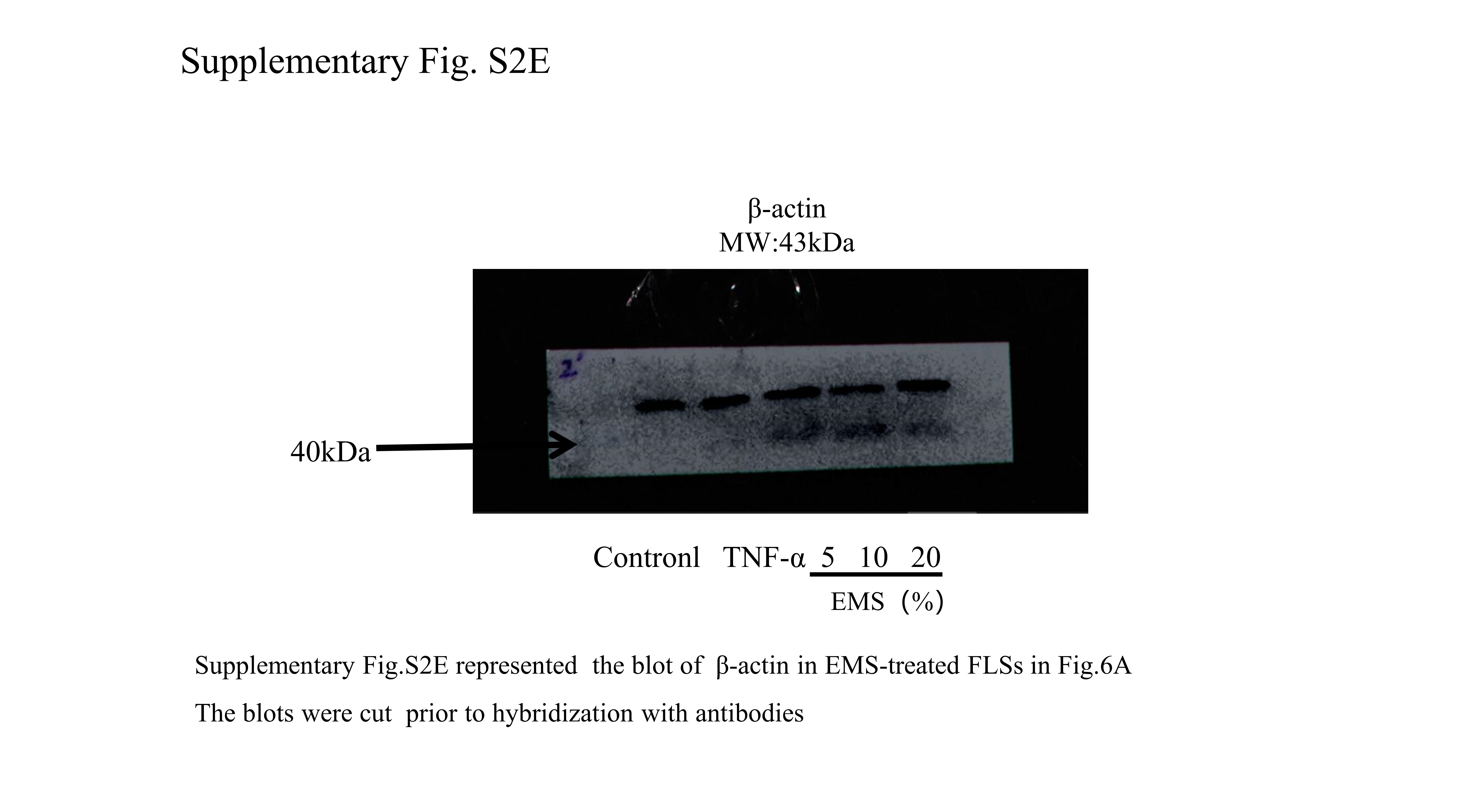

Supplement: Supplementary file 3 — Supplementary Material 3. [file 12906_2026_5359_MOESM3_ESM.docx]

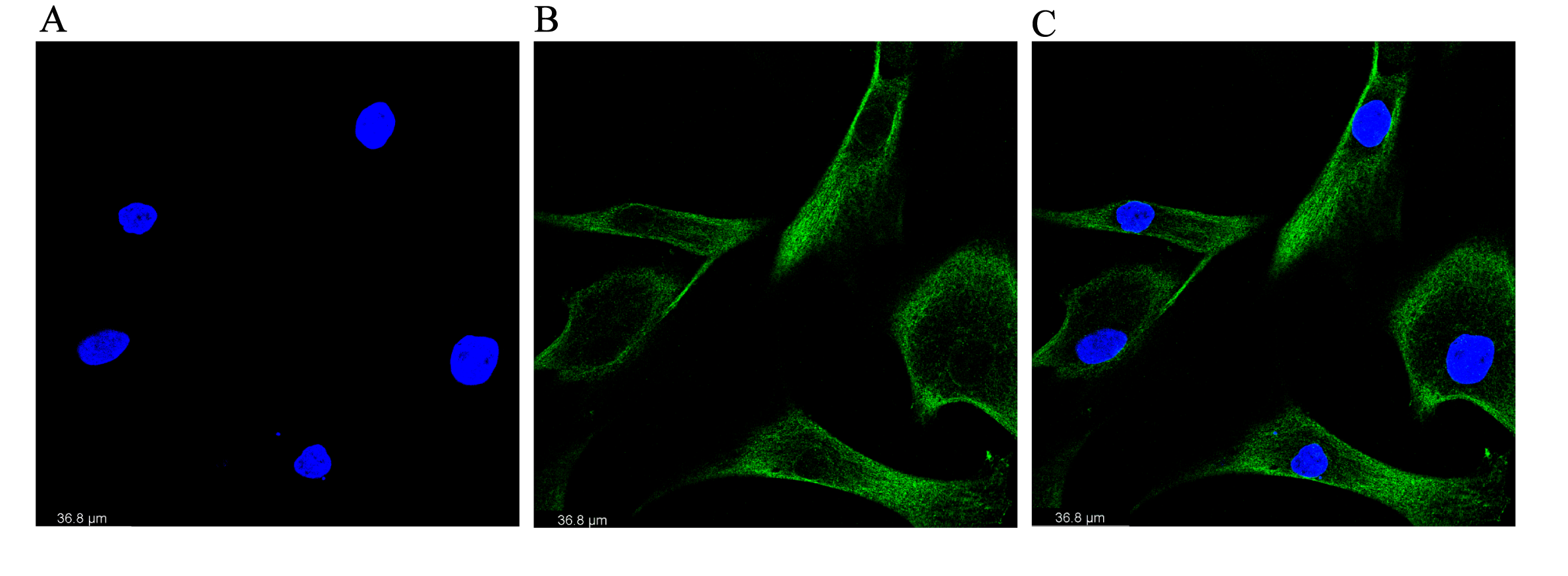

Supplement: Supplementary file 4 — Supplementary Material 4. [file 12906_2026_5359_MOESM4_ESM.tif]
